# Sex-specific DNA methylation differences in Alzheimer’s disease pathology

**DOI:** 10.1186/s40478-021-01177-8

**Published:** 2021-04-26

**Authors:** Lanyu Zhang, Juan I. Young, Lissette Gomez, Tiago C. Silva, Michael A. Schmidt, Jesse Cai, Xi Chen, Eden R. Martin, Lily Wang

**Affiliations:** 1 Division of Biostatistics, Department of Public Health Sciences, University of Miami, Miller School of Medicine, Miami, FL 33136 USA; 2 Dr. John T Macdonald Foundation Department of Human Genetics, University of Miami, Miller School of Medicine, Miami, FL 33136 USA; 3 John P. Hussman Institute for Human Genomics, University of Miami, Miller School of Medicine, Miami, FL 33136 USA; 4 Brentwood High School, 5304 Murray Ln, Brentwood, TN 37027 USA; 5 Sylvester Comprehensive Cancer Center, University of Miami, Miller School of Medicine, Miami, FL 33136 USA

**Keywords:** Alzheimer’s disease, Sex-specific, DNA methylation, Epigenome-wide association study

## Abstract

Sex is an important factor that contributes to the clinical and biological heterogeneities in Alzheimer’s disease (AD), but the regulatory mechanisms underlying sex disparity in AD are still not well understood. DNA methylation is an important epigenetic modification that regulates gene transcription and is known to be involved in AD. We performed the first large-scale sex-specific meta-analysis of DNA methylation differences in AD neuropathology, by re-analyzing four recent epigenome-wide association studies totaling more than 1000 postmortem prefrontal cortex brain samples using a uniform analytical pipeline. For each cohort, we employed two complementary analytical strategies, a sex-stratified analysis that examined methylation-Braak stage associations in male and female samples separately, and a sex-by-Braak stage interaction analysis that compared the magnitude of these associations between different sexes. Our analysis uncovered 14 novel CpGs, mapped to genes such as *TMEM39A* and *TNXB* that are associated with the AD Braak stage in a sex-specific manner. *TMEM39A* is known to be involved in inflammation, dysregulated type I interferon responses, and other immune processes. *TNXB* encodes tenascin proteins, which are extracellular matrix glycoproteins demonstrated to modulate synaptic plasticity in the brain. Moreover, for many previously implicated genes in AD neuropathology, such as *MBP* and *AZU1*, our analysis provided the new insights that they were predominately driven by effects in only one sex. These sex-specific DNA methylation differences were enriched in divergent biological processes such as integrin activation in females and complement activation in males. Our study implicated multiple new loci and biological processes that affected AD neuropathology in a sex-specific manner.

**Supplementary Information:**

The online version contains supplementary material available at 10.1186/s40478-021-01177-8.

## Introduction

Late-onset Alzheimer’s disease (LOAD) is the most common cause of dementia. With the aging population in the U.S., Alzheimer’s disease (AD) has become a major public health concern and one of the most financially costly diseases [1]. Almost two-thirds of AD patients in the U.S. are women [2]. After diagnosis, women also progress faster with more rapid cognitive and functional declines [3–8]. On the other hand, it has also been reported men with AD have an increased risk for death [9–11]. However, the molecular mechanisms underlying these observed disparities in AD are still not well understood. Previous studies have shown that epigenetics is an important contributor to the sex differences in brain functions and vulnerability to diseases [12–16]. Among epigenetic modifications, DNA methylation profiles differ significantly between males and females at many loci in adult brains [17]. Importantly, alterations of DNA methylation levels have also been implicated in multiple neurological disorders including AD [18–22].

However, thus far, a comprehensive characterization for the contribution of sex to DNA methylation differences in AD neuropathology has not been performed. In the identification of sex-specific effects, statistical power is a major challenge [23]. Stratifying by sex reduces the sample size of both groups. Also, comparing methylation to disease associations between the sexes by testing the interaction effect would require a much larger sample size than detecting the main effect with the same magnitude [24]. To address these challenges, we performed a comprehensive meta-analysis of more than 1000 post-mortem brain prefrontal cortex (PFC) samples, collected from four recent AD epigenome-wide association studies [18–21], to identify the most consistent DNA methylation differences affected by AD neuropathology in a sex-specific manner. Within each cohort, to identify sex-specific differences in AD neuropathology, we employed two complementary approaches, a sex-stratified analysis that examined methylation-Braak stage associations in female and male samples separately, and a sex-by-Braak stage interaction analysis that compared the magnitude of these associations between different sexes. As sex is a strong factor in driving inter-personal variabilities in AD, the results of this study are particularly relevant for precision medicine.

## Methods

### Study cohorts

Our meta-analysis included 1,030 prefrontal cortex brain samples (642 female samples and 388 male samples) from four independent cohorts (Additional file 2: Table S1), previously described in the ROSMAP [18], Mt. Sinai [20], London [19], and Gasparoni [21] DNA methylation studies.

### Pre-processing of DNA methylation data

All the samples in this study were measured using the Infinium HumanMethylation450 BeadChip. Additional file 2: Table S2 shows the number of CpGs and samples removed at each quality control step. Quality control for CpG probes included removing probes with detection P-value < 0.01 in all samples of a cohort using the minfi R package, removing the 2623 CpGs associated with cigarette smoking identified in Joehanes et al. [25] (at P-value < 1 × 10^–7^ threshold), and removing CpGs having a single nucleotide polymorphism (SNP) with minor allele frequency (MAF) $\ge 0.01$ present in the last 5 base pairs of the probe using the DMRcate R package (with function rmSNPandCH and option dist = 5, mafcut = 0.01). For the quality control of samples, we removed samples with low bisulfite conversion efficiency (i.e., < 88%) or detected as outliers in principal component analysis (PCA). More specifically, PCA was performed using the 50,000 most variable CpGs for each cohort. Samples that were within ± 3 standard deviations from the means of PC1 and PC2 were selected to be included in the final sample set. The quality-controlled methylation datasets were next subjected to the QN.BMIQ normalization procedure as recommended by a recent systematic study of different normalization methods [26]. More specifically, we first applied quantile normalization as implemented in the lumi R package to remove systematic effects between samples. Next, we applied the β-mixture quantile normalization (BMIQ) procedure [27] as implemented in the wateRmelon R package [28] to normalize beta values of type 1 and type 2 design probes within the Illumina arrays. Finally, to remove batch effects, we applied the linear model methylation M value ~ methylation slide to M values of each CpG. The methylation residuals from these linear models were then used for subsequent analysis.

### Single cohort analysis

To identify sex-specific DNA methylation differences in AD neuropathology, we performed both a sex-stratified analysis and a sex-by-Braak stage interaction analysis for each of the four brain sample cohorts. In the sex-stratified analysis, we tested methylation-Braak stage associations in female and male samples separately. In sex-by-Braak stage interaction analysis, we analyzed both female and male samples simultaneously and compared slopes for methylation-Braak stage associations in females and males.

More specifically, in the sex-stratified analysis, for each CpG, we applied the model methylation residuals ~ age at death + Braak stage + estimated neuron proportions to female samples and male samples separately, where methylation residuals were obtained in “Pre-processing of DNA methylation data” described above. Here, the neuron proportion for each sample was estimated using the CETS R package [29], an R software that quantifies neuronal proportions from DNA methylation data using cell epigenotype specific (CETS) marks. In sex-by-Braak stage interaction analysis, for each CpG, we applied the model methylation residuals ~ age at death + sex + Braak stage + sex*Braak stage + sex*age at death + estimated neuron proportions to samples including both sexes.

For the analysis of differentially methylated regions (DMRs), we used the coMethDMR R package [30] to analyze 40,010 pre-defined genomic regions on the Illumina 450 k arrays and identify co-methylated DMRs associated with Braak stage. The pre-defined genomic regions are regions on the Illumina array covered with clusters of contiguous CpGs where the maximum separation between any two consecutive probes is 200 base pairs. First, coMethDMR selects *co-methylated regions* within these pre-defined contiguous genomic regions. Next, we summarized methylation M values within these co-methylated regions using medians and tested them against the AD Braak stage. The same linear models described for the analysis of CpGs were then applied to the median value of each DMR. We considered CpGs (or DMRs) with a false discovery rate (FDR) less than 0.05 in female samples or male samples to be significant.

### Inflation assessment and correction

For genome-wide discoveries to be valid, the false positive rate of the study should be properly controlled. Because systematic inflation of test statistics can lead to an increase in the number of false-positive results, traditionally genomic inflation factor [31] is typically used to quantify the amount of inflation in genome-wide association studies (GWAS) of genetic variants. However, as shown by simulation studies [32], real datasets [32], and theory [31], the conventional genomic inflation factor (lambda or $\lambda$ used interchangeably below) is dependent on the expected number of true associations. Because in a typical epigenome-wide association study (EWAS), it is expected that small effects from many CpGs might be associated with the phenotype, the genomic inflation factor would overestimate actual test-statistic inflation. To estimate genomic inflations more accurately in EWAS, Iterson et al. [32] developed a Bayesian method that estimates inflation in EWAS based on empirical null distributions, which is implemented in the Bioconductor package bacon.

In this study, to assess inflation of the test statistics, we used quantile–quantile (QQ) plots and estimated genomic inflation factors using both the conventional approach and the *bacon* method [32] (Additional file 1: Fig. S1). The *bacon* method was also used to obtain inflation-corrected effect sizes, standard errors, and P-values for each cohort, which were then combined by inverse-variance weighted meta-analysis models using R package meta.

### Meta-analysis

The evidence for heterogeneity of study effects was tested using Cochran’s Q statistic [33]. The inverse-variance weighted fixed effects model was first applied to synthesize statistical significance from individual cohorts. Even though the fixed effects model for meta-analysis does not require the assumption of homogeneity [34], for the CpGs (or genomic regions) with nominal evidence for heterogeneity (nominal P_heterogeneity_ < 0.05), we also applied random effects meta-analysis [35] and assigned final meta-analysis P-value based on the random effects model. For each CpG (and for each genomic region), we used the R package meta to obtain Braak stage effect in female samples and male samples separately in sex-stratified analysis, as well as meta-analysis P-values for sex-by-Braak stage interaction.

### Identifying sex-specific differences

In the sex-stratified analysis, we selected significant CpGs (or genomic regions) with FDR < 0.05 in female samples or male samples separately. In sex-by-Braak stage interaction analysis, because the standard error of interaction effect sex × Braak stage is typically much larger than those for main Braak stage effects, the conventional approach for controlling false discovery rate often results in low power for discovering interaction effects [36]. Therefore, we used a stagewise analysis approach, previously proposed by van de Berge et al. (2017) [36], to help improve power in high-throughput experiments where multiple hypotheses are tested for each gene. More specifically, in the *screening step*, for each CpG (or genomic region), we tested the global null hypothesis that there is methylation-Braak stage association in either male or female samples. Next, in the *confirmation step*, we considered three individual null hypotheses for each CpG (or DMR): (a) there is no methylation-Braak stage association in male samples, (b) there is no methylation-Braak stage association in female samples, and (c) the methylation-Braak stage associations in male samples and female samples are the same. For the CpGs (or genomic region) selected in the screening step, these three individual hypotheses were then tested while controlling family-wise error rate (FWER) as described in van de Berge et al. (2017)[36].

The stagewise analysis described above was implemented using the stageR package to identify CpGs (or genomic regions) with significant differential methylation—Braak stage associations in females and males. In the screening step, we considered meta-analysis P-values for the Braak stage in female samples and male samples (p.meta.female, p.meta.male), and used the minimum of these two meta-analysis P-values to represent each CpG (or genomic region). In the confirmation step, the parameter pConfirmation was defined using three P-values for each CpG (or genomic region): p.meta.female, p.meta.male, and p.meta.interaction (meta-analysis P-value for sex × Braak stage).

### Enrichment and pathway analysis

The probes on the Illumina 450k array are annotated according to their locations with respect to genes (TSS1500, TSS200, 5′UTR, 1stExon, gene body, 3′UTR, intergenic) or CpG islands (island, shore, shelf, open sea). To understand the genomic context of sex-specific DNA methylation differences in AD neuropathology, we compared the FDR significant methylation differences from sex-stratified analysis with different types of genomic features. As Braak-associated methylation differences can occur at both significant individual CpGs and significant DMRs, we considered the CpGs located at significant individual CpGs or within significant DMRs jointly in this analysis, by testing their over- and under-representation in different types of genomic features using Fisher’s exact test. More specifically, the proportion of significant CpGs mapped to a particular type of genomic feature (e.g., CpG islands) (foreground) was compared to the proportion of CpGs on the array that mapped to the same type of genomic feature (background).

Also, we used Fisher’s test to assess enrichment of significant CpGs and DMRs in different chromatin states by comparing with the 15-chromatin state data for bulk PFC tissue samples (E073) from the Roadmap Epigenomics Project [37]. Using combinations of histone modification marks, ChromHMM [38] was previously used to annotate segments of the genome with different chromatin states (repressed, poised, and active promoters, strong and weak enhancers, putative insulators, transcribed regions, and large-scale repressed and inactive domains), which were shown to vary across sex, tissue type, and developmental age [39]. Similarly, we tested enrichment of significant CpGs and DMRs in binding sites of transcription factors and chromatin proteins from the ENCODE project [40] and CODEX database [41] using Locus Overlap Analysis as implemented in the LOLA R package [42].

Finally, we performed pathway analysis by comparing the genes with significant DNA methylation differences in AD neuropathology (identified in sex-stratified analysis) with the canonical pathways and biological process GO terms in MSigDB using Gene Set Enrichment Analysis (GSEA) [43]. First, we linked each CpG and each pre-defined genomic region tested in DMR analysis to genes by annotating them using the GREAT (Genomic Regions Enrichment of Annotations Tool) software, which associates genomic regions to target genes. With the default “Basal plus method”, GREAT links each gene to a regulatory region consisting of a basal domain that extends 5 kb upstream and 1 kb downstream from its transcription start site, and an extension up to the basal regulatory region of the nearest upstream and downstream genes within 1 Mb [44]. Next, we represented each gene by the smallest P-value if there are multiple CpGs or genomic regions associated with them. To remove selection bias due to different numbers of CpGs or genomic regions associated with each gene (i.e., the smallest P-value for a gene with many CpGs or genomic regions linked to it is likely to be smaller than the smallest P-value for a gene with only a few linked CpGs or genomic regions), we next fit a generalized additive model [45] using the R package mgcv: ${Y}_{i}\sim f({n.links}_{i})$ where *Y*_*i*_ is negative log (base 10) transformation of the smallest P-value for gene *i* in the analysis of female samples (or male samples), *n.links*_*i*_ is the number of CpGs or genomic regions linked to gene *i,* and *f* is a penalized spline function. We assumed gamma distribution for *Y*_*i*_, as under the null hypothesis of no association, *Y*_*i*_ follows the chi-square distribution (a special case of gamma distribution). The residuals from this model were estimated and used to generate a ranked gene list, which was then used as input for GSEA (in pre-ranked mode) to identify canonical pathways and gene ontology terms (MsigDB C2:CP and C5:BP collections of gene sets) enriched with significant methylation differences in female samples and male samples separately.

### Integrative methylation and gene expression analysis

To systematically evaluate transcriptional differences near the observed sex-specific DNA methylation differences, we next performed integrative methylation—gene expression analysis using data on 333 female samples and 196 male samples from the ROSMAP study with matched DNA methylation and RNA-seq gene expression profiles measured on the prefrontal cortex. To this end, normalized FPKM (Fragments Per Kilobase of transcript per Million mapped reads) gene expression values for the ROSMAP study were downloaded from the AMP-AD Knowledge Portal (Synapse ID: syn3388564).

First, we linked significant CpGs (or DMRs) to nearby genes using GREAT [44], which associates genomic regions to target genes. Next, we removed confounding effects in DNA methylation data by fitting the model methylation M value ~ neuron.proportion + batch + sample.plate + ageAtDeath and extracting residuals from this model; these are the *ROSMAP methylation residuals*. Similarly, we also removed potential confounding effects in RNA-seq data by fitting model log2(normalized FPKM values + 1) ~ ageAtDeath + markers for cell types. The last term, “markers for cell types,” included multiple covariate variables to adjust for the multiple types of cells in the brain samples. More specifically, we estimated expression levels of genes that are specific for the five main cell types present in the CNS: ENO2 for neurons, GFAP for astrocytes, CD68 for microglia, OLIG2 for oligodendrocytes, and CD34 for endothelial cells, and included these as variables in the above linear regression model, as in previous large study of AD samples [18]. The residuals extracted from this model are the *ROSMAP gene expression residuals*.

Finally, for each gene expression and CpG (or DMR) pair, we then tested the association between gene expression residuals and methylation residuals using a linear model: ROSAMP gene expression residuals ~ ROSMAP methylation residuals + Braak stage. For significant DMRs, this analysis was repeated, except that methylation M value was replaced with median methylation M value from multiple CpGs in the DMR.

### Sex-specific mQTL analysis

To identify methylation quantitative trait loci (mQTLs) for the significant DMRs and CpGs, we tested associations between the methylation levels with nearby SNPs, using the ROSMAP study dataset, which had matched genotype data and DNA methylation data for 434 female samples and 254 male samples. The ROSMAP genotype data was downloaded from AMP-AD (syn3157325) and imputed to the Haplotype Reference Consortium r1.1 reference panel [46]. There were two batches of genotype data, measured by Affymetrix GeneChip 6.0 (Affymetrix, Inc, Santa Clara, CA, USA) and Illumina HumanOmniExpress (Illumina, Inc, San Diego, CA, USA).

The male samples and female samples were analyzed separately. To reduce the number of tests, we focused on identifying *cis* mQTLs located within 500 kb from the start or end of the DMR (or position of the significant CpG) [47]. We additionally required SNPs to (1) have a minor allele frequency of at least 1%, (2) be imputed with good certainty: information metric (info score) $\ge$ 0.4, and (3) be associated with AD case–control status (as determined by clinical consensus diagnosis of cognitive status), after adjusting for age, batch, and the first 3 PCs estimated from genotype data, at nominal P-value less than 0.05. Next, for the ROSMAP methylation residuals obtained in section “Integrative methylation—gene expression analysis”, we fit the linear model ROSMAP methylation residual ~ SNP dosage + batch + PC1 + PC2 + PC3, where PC1, PC2, and PC3 are the first three PCs estimated from genotype data, to test the association between methylation residuals in CpGs and the imputed allele dosages for SNPs to identify mQTLs. The analysis for DMRs was the same except that we replaced ROSMAP methylation residual with median (ROSMAP methylation residuals) of all CpGs located within the DMR. SNPs with FDR less than 0.05 in the linear model described above were considered to be significant mQTLs.

### Drug target analysis

We compared our list of sex-specific DNA methylation differences with targets of drugs prescribed to AD patients or in the development for AD in the ChEMBL database [48] (https://www.ebi.ac.uk/chembl/). To this end, we overlapped genes mapped to significant CpGs or DMRs with the genes targeted by compounds annotated to “Alzheimer Disease” in ChEMBL.

## Results

### Description of EWAS cohorts and data

Among the four cohorts (Additional file 2: Table S1), the mean age at death ranged from 79.3 to 87.2 years in females and from 67.5 to 85.0 years in males. The number of CpGs and samples removed at each quality control step are presented in Additional file 2: Table 2. For females, inflation factor lambdas ($\lambda )$ by the conventional approach ranged from 1.060 to 1.154, and lambdas based on the *bacon* approach [32] (${\lambda }_{bacon}$) ranged from 1.021 to 1.059 (Additional file 1: Fig. S1). Similarly, for males, $\lambda$ ranged from 0.906 to 1.265, and ${\lambda }_{bacon}$ ranged from 0.957 to 1.114. These values are comparable to those obtained in other recent large-scale EWAS [49].

### Sex-specific DNA methylation differences in AD neuropathology

**Fig. 1:**
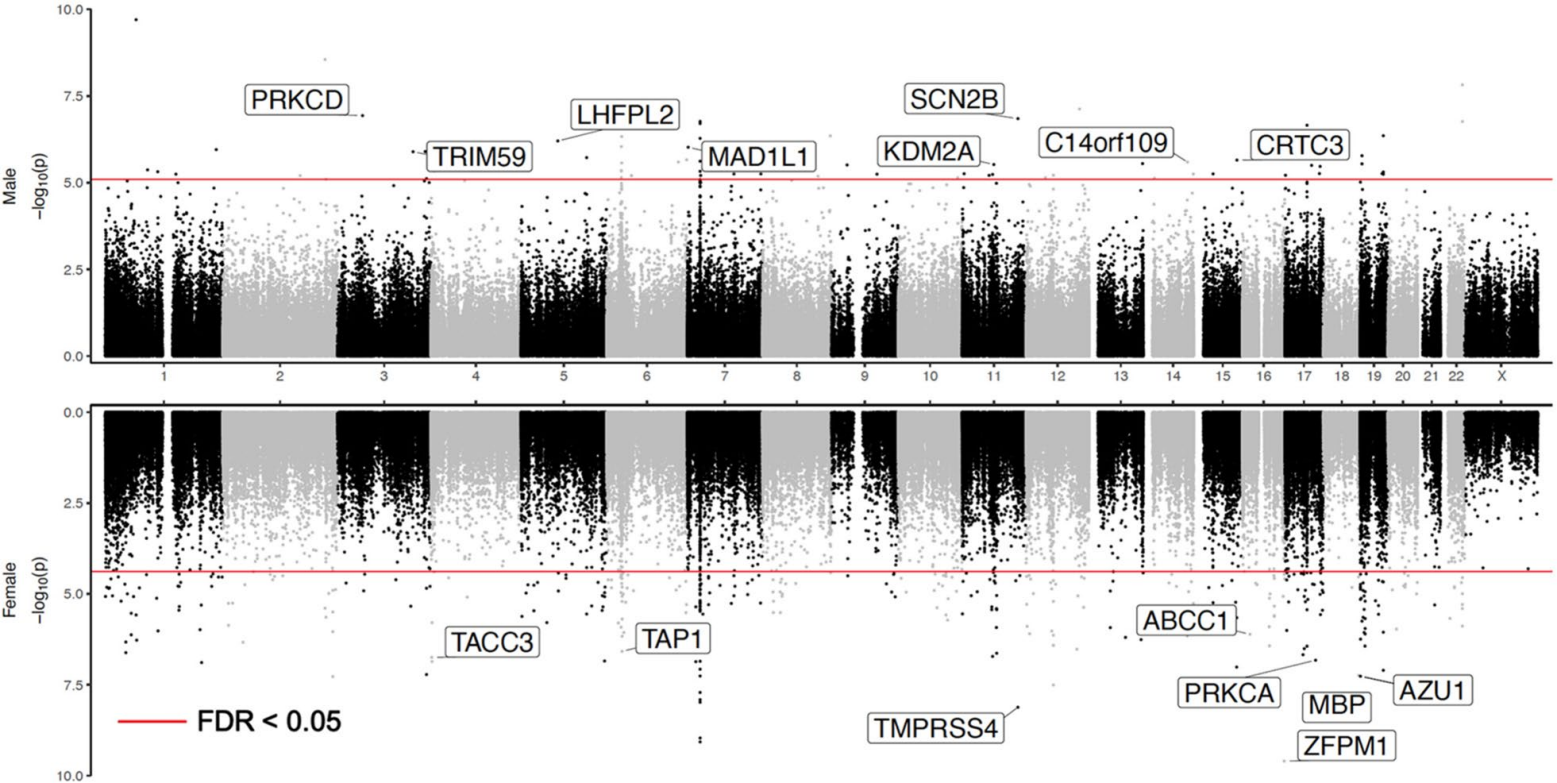
Miami plot of sex‐stratified analysis results. The X‐axis are chromosome numbers. The Y‐axis show –log_10_(P-value) of CpG – Braak stage associations in males (above X‐axis) or in females (below X‐axis). The genes corresponding to top 10 CpGs that are the most significant in one sex (FDR < 0.05), but not significant in another sex (P‐value > 0.05) are highlighted

In the sex-stratified analysis, at 5% FDR, our meta-analysis identified 381 and 76 CpGs, mapped to 245 and 51 genes in female and male samples, respectively (Fig. 1, Table 1, Additional file 2: Tables S3, S4). Similarly, at 5% FDR, we also identified 72 and 27 DMRs, mapped to 66 and 22 genes, in female and male samples, respectively (Table 2, Additional file 2: Tables S5, S6). Among them, 3.6% (16 out of 441 unique FDR-significant CpGs) and 12.5% (11 out of 88 unique FDR-significant DMRs) were significant in both females and males with the same direction of change. The average number of CpGs per DMR was 6.5 ± 8.9. Also, the FDR-significant methylation differences at CpGs and DMRs did not completely overlap. Only 89 out of the 381 (23.4%) significant CpGs in females, and 13 out of the 76 (17.1%) significant CpGs in males overlapped with the significant DMRs. Among all CpGs and all DMRs, the effect estimates in males and females correlated only modestly (*r*_CpG_ = 0.124, *r*_DMR_ = 0.170, Additional file 1: Fig. S2), and about half (53% of CpGs, 54% of DMRs) were in the same direction of change in males and females, similar to what would be expected by chance.

**Table 1:** Top 10 CpGs in sex-stratified analysis. Shown are CpGs that are highly significant in one sex (FDR < 0.05), but not significant (P-value > 0.05) in the other sex. For each CpG, annotations include the location of the CpG based on hg19/GRCh37 genomic annotation (chr, position), Illumina gene annotations, nearby genes based on GREAT, and chromatin state. The inverse-variance weighted meta-analysis regression model results include estimated effect size (estimate) where CpGs that are hyper-methylated in samples with AD neuropathology have positive values, and its associated standard error (se), P-value, and false discovery rate (FDR) for multiple comparison corrections. Bold indicates a significant association at 5% FDR

cpg	Annotations					Female samples				Males samples			
	chr	Position	Illumina	GREAT (distance to TSS)	chromatin state	estimate	se	P-value	FDR	estimate	se	P-value	FDR
*Significant only in female samples*													
cg09502865	chr16	88600155	ZFPM1	ZC3H18(− 36633); ZFPM1(+ 80431)	Active TSS	0.179	0.028	**2.50E−10**	**1.15E−04**	0.094	0.034	4.46E−01	9.64E−01
cg05235171	chr11	117958104	TMPRSS4	TMPRSS4(+ 10301); SCN4B(+ 65430)	Quiescent/Low	− 0.168	0.029	**7.61E−09**	**6.99E−04**	− 0.025	0.033	4.47E−01	9.64E−01
cg15610437	chr19	827821	AZU1	AZU1(− 4)	Weak Repressed PolyComb	− 0.155	0.029	**5.45E−08**	**1.99E−03**	− 0.090	0.034	9.79E−02	8.25E−01
cg13572782	chr18	74799495	MBP	MBP(+ 45229); ZNF236(+ 263380)	Active TSS	0.157	0.029	**5.75E−08**	**1.99E−03**	0.047	0.036	1.90E−01	8.92E−01
cg17881200	chr7	27138850		HOXA1(− 3258)	Repressed PolyComb	0.152	0.029	**1.41E−07**	**2.98E−03**	0.131	0.034	6.22E−02	7.73E−01
cg22632947	chr17	64787784	PRKCA	CACNG5(− 43450); PRKCA(+ 488841)	Flanking Active TSS	− 0.139	0.026	**1.50E−07**	**3.00E−03**	− 0.005	0.028	8.57E−01	9.95E−01
cg15467503	chr4	1727234	TACC3	TMEM129(− 4177); TACC3(+ 3969)	Weak transcription	− 0.146	0.028	**1.81E−07**	**3.45E−03**	− 0.083	0.034	1.33E−01	8.59E−01
cg26033526	chr6	32819858	TAP1	PSMB9(− 2079); TAP1(+ 1896)	Genic enhancers	0.148	0.029	**2.64E−07**	**4.18E−03**	− 0.021	0.034	5.23E−01	9.73E−01
cg08363067	chr16	16170085	ABCC1	ABCC1(+ 126652); ABCC6(+ 147235)	Weak transcription	− 0.120	0.024	**7.68E−07**	**7.32E−03**	− 0.050	0.028	7.41E−02	7.94E−01
cg12926693	chr6	36665611		RAB44(− 17644); CDKN1A(+ 19125)	Quiescent/Low	− 0.149	0.030	**8.59E−07**	**7.32E−03**	− 0.067	0.035	5.19E−02	7.52E−01
*Significant only in male samples*													
cg07687398	chr3	53198666	PRKCD	PRKCD(+ 3531); TKT(+ 91371)	Weak transcription	0.052	0.030	8.63E−02	8.19E−01	0.183	0.035	**1.16E−07**	**9.88E−03**
cg10513118	chr11	118047203	SCN2B	SCN2B(+ 184)	Active TSS	0.038	0.030	2.01E−01	9.17E−01	0.172	0.033	**1.42E−07**	**9.88E−03**
cg21253952	chr8	143662999		ARC(+ 33833); BAI1(+ 132209)	Weak Repressed PolyComb	− 0.019	0.030	5.33E−01	9.84E−01	0.169	0.034	**4.44E−07**	**1.53E−02**
cg18281939	chr5	77783895	LHFPL2	SCAMP1(+ 127557); LHFPL2(+ 160752)	Weak transcription	− 0.022	0.029	4.55E−01	9.76E−01	− 0.179	0.036	**6.26E−07**	**1.79E−02**
cg11809272	chr6	31409361		HLA-B(− 84398); MICB(− 56530)	Quiescent/Low	− 0.001	0.031	9.77E−01	1.00E + 00	0.161	0.033	**9.08E−07**	**2.17E−02**
cg15952933	chr7	1899886	MAD1L1	ELFN1(+ 172132); MAD1L1(+ 372991)	Weak transcription	− 0.058	0.033	7.56E−02	8.00E−01	− 0.156	0.032	**9.48E−07**	**2.17E−02**
cg11614451	chr3	160167729	TRIM59	ENSG00000248710(− 113); TRIM59(− 113)	Flanking Bivalent TSS/Enh	− 0.041	0.029	1.56E−01	8.92E−01	− 0.150	0.031	**1.28E−06**	**2.44E−02**
cg18942110	chr15	91072797	CRTC3	CRTC3(− 502)	Active TSS	0.031	0.030	3.06E−01	9.52E−01	− 0.164	0.035	**2.23E−06**	**3.19E−02**
cg01655008	chr14	93652954	C14orf109	MOAP1(− 1687); TMEM251(+ 1597)	Transcr. at gene 5′ and 3'	− 0.042	0.029	1.51E−01	8.89E−01	− 0.152	0.032	**2.54E−06**	**3.33E−02**
cg02331272	chr11	66964208	KDM2A	ADRBK1(− 69672); KDM2A(+ 77051)	Weak transcription	0.058	0.030	1.10E−01	8.52E−01	− 0.166	0.036	**2.99E−06**	**3.52E−02**

**Table 2:** Top 10 and 6 DMRs in sex-stratified analysis. Shown are DMRs that are highly significant in one sex (FDR < 0.05), but not significant (P-value > 0.05) in another sex. Only 6 DMRs satisfied these criteria in male samples. For each DMR, annotations include the location of the DMR based on hg19/GRCh37 genomic annotation (DMR), Illumina gene annotations, nearby genes based on GREAT, and chromatin state. The inverse-variance weighted meta-analysis regression models results based on coMethDMR include estimated effect size (estimate) where DMRs that are hyper-methylated in samples with AD neuropathology have positive values, its associated standard error (se), P-value, and false discovery rate (FDR) for multiple comparison corrections. Shown in bold are significant results with FDR < 0.05

DMR	Annotations			Female samples				Male samples			
	Illumina	GREAT (distance to TSS)	chromatin state	estimate	se	P-value	FDR	estimate	se	P-value	FDR
*Significant only in female samples*											
chr5:27038605-27038836	CDH9	CDH9(− 28)	Active TSS;Flanking Active TSS	0.128	0.024	**1.87E−07**	**2.30E−03**	0.077	0.027	2.74E−01	8.99E−01
chr15:31621629-31621843	KLF13	KLF13(+ 2678); OTUD7A(+ 325806)	Active TSS	0.115	0.023	**3.30E−07**	**2.30E−03**	0.042	0.027	1.19E−01	8.07E−01
chr18:74799495-74799572	MBP	MBP(+ 45191); ZNF236(+ 263418)	Active TSS	0.138	0.028	**5.81E−07**	**2.48E−03**	0.049	0.033	1.30E−01	8.13E−01
chr7:45066738-45067057	CCM2	CCM2(+ 273)	Weak transcription	− 0.125	0.026	**2.23E−06**	**6.20E−03**	− 0.090	0.027	5.51E−02	7.00E−01
chr13:67803895-67804060	PCDH9	PCDH9(+ 490)	Active TSS	− 0.088	0.019	**5.04E−06**	**1.02E−02**	− 0.017	0.022	4.40E−01	9.50E−01
chr16:66969401-66969506	CES2;FAM96B	FAM96B(− 1151); CES2(+ 1091)	Flanking Active TSS	0.115	0.025	**5.68E−06**	**1.02E−02**	0.052	0.028	6.52E−02	7.28E−01
chr15:40268421-40268777	EIF2AK4	EIF2AK4(+ 42245); SRP14(+ 62790)	Active TSS	0.125	0.028	**7.12E−06**	**1.16E−02**	− 0.005	0.032	8.72E−01	9.94E−01
chr5:14492774-14492945	TRIO	FAM105B(− 171997); TRIO(+ 349049)	Strong transcription	− 0.103	0.023	**8.25E−06**	**1.24E−02**	− 0.009	0.029	7.49E−01	9.85E−01
chr17:56736571-56737118	TEX14	SEPT4(− 118666); TEX14(+ 32539)	Active TSS	− 0.070	0.016	**9.13E−06**	**1.26E−02**	0.017	0.017	3.20E−01	9.16E−01
chr17:46651722-46651952	HOXB3	HOXB3(− 20553); HOXB4(+ 5636)	Repressed PolyComb	0.113	0.025	**9.24E−06**	**1.26E−02**	0.030	0.027	2.63E−01	8.97E−01
*Significant only in male samples*											
chr17:1173700-1173767	BHLHA9	BHLHA9(− 119)	Weak Repressed PolyComb	− 0.041	0.025	1.07E−01	8.23E−01	− 0.118	0.026	**5.08E−06**	**1.59E−02**
chr11:68780866-68780984	MRGPRF	MRGPRF(− 48)	Flanking Bivalent TSS/Enh	0.032	0.024	1.80E−01	8.93E−01	0.112	0.026	**1.16E−05**	**2.96E−02**
chr12:52437299-52437571		C12orf44(− 26320); NR4A1(+ 20819)	Enhancers	0.009	0.017	5.93E−01	9.93E−01	0.075	0.017	**1.38E−05**	**3.31E−02**
chr14:89017615-89017776	PTPN21	PTPN21(+ 3381); SPATA7(+ 165822)	Active TSS	0.046	0.024	6.03E−02	7.31E−01	0.102	0.024	**1.46E−05**	**3.31E−02**
chr19:17691465-17691691	GLT25D1	COLGALT1(+ 25175); UNC13A(+ 107430)	Strong transcription	− 0.006	0.022	8.06E−01	1.00E + 00	0.107	0.025	**2.47E−05**	**4.16E−02**
chr6:32920567-32921233	HLA-DMA	ENSG00000248993(− 1); HLA− DMA(− 1)	Flanking Active TSS; Active TSS; Quiescent/Low	− 0.045	0.024	3.16E−01	9.56E−01	− 0.117	0.028	**3.17E−05**	**4.78E−02**

In sex-by-Braak stage interaction analysis, we identified significant interaction at 14 CpGs, but no significant interactions at DMRs at 5% FDR. There was also little overlap between significant DNA methylation differences identified in sex-stratified and sex-by-Braak stage interaction analyses. Only 4 CpGs were identified by both analyses (Table 3). To understand this discrepancy, note that the sex-stratified analysis detected many differences that are attenuated but might be in the same direction in one sex group compared to the other. More specifically, among the FDR-significant CpGs identified in the sex-stratified analysis, many of them (370 out of 381 significant CpGs in the analysis of female samples, and 65 out of 76 significant CpGs in the analysis of male samples) had the same direction of change for methylation-Braak stage association in both sexes (Additional file 2: Tables S3, S4). In Table 1, among the 10 most significant CpGs from the sex-stratified analysis, 9 female-specific and 6 male-specific CpGs had the same direction of methylation-Braak stage association in both sexes. On the other hand, in sex-by-Braak stage interaction analysis, 13 out of the 14 significant CpGs had the opposite directions of differences for methylation-Braak stage associations in females and males (Table 3). Therefore, the interaction analysis was able to identify CpGs with large differences in sex-specific effect estimates, often in different directions, but these effects might not have reached FDR significance in sex-stratified analysis. For example, in Table 3, the CpG with the most significant interaction (cg13212831) had effect estimates of 0.083 and − 0.139 for females and males, respectively. In sex-stratified analysis, although the methylation-Braak stage associations were highly significant (P-value_female_ = 0.006, P-value_male_ = 4.1 × 10^–5^), they did not reach 5% FDR significance threshold (FDR_female_ = 0.413, FDR_male_ = 0.097). Therefore, the results from sex-stratified analysis and sex-by-Braak stage interaction analysis complemented each other.

**Table 3:** Results from sex-by-Braak stage interaction analysis. For each CpG, annotations include the location of the CpG based on hg19/GRCh37 genomic annotation (chr, position), Illumina gene annotations, nearby genes based on GREAT, and chromatin state. The inverse-variance weighted meta-analysis regression model results include estimated effect size (estimate) where CpGs that are hyper-methylated in samples with AD neuropathology have positive values, its associated standard error (se), and P-value. Adj.pval is adjusted P-value from stageR analysis. Shown in bold are significant results (adj.pval < 0.05 for interaction and P-value < 0.05 for one sex). * indicates these CpGs also reached 5% FDR significance in sex-stratified analysis

cpg	Annotations					Female samples			Male samples			Sex * Braak stage interaction			
	chr	Position	Illumina	GREAT (distance to TSS)	**chromatin state**	**estimate**	**se**	**P-value**	**estimate**	**se**	**P-value**	**estimate**	**se**	**P-value**	**adj.pval**
cg13212831	chr1	56046343		USP24(-365558); PPAP2B(+ 998897)	Weak transcription	0.083	0.030	**6.15E-03**	− 0.139	0.034	**4.10E-05**	− 0.222	0.044	**6.38E-07**	**3.54E-04**
cg25734825	chr3	119182523	TMEM39A	TMEM39A(+ 5)	Active TSS	0.059	0.030	5.01E-02	− 0.156	0.036	**1.21E-05**	− 0.220	0.045	**1.12E-06**	**6.22E-04**
*cg02331272	chr11	66964208	KDM2A;KDM2A	ADRBK1(-69672); KDM2A(+ 77051)	Weak transcription	0.058	0.030	1.10E-01	− 0.166	0.036	**2.99E-06**	− 0.215	0.046	**2.25E-06**	**1.25E-03**
cg10622825	chr1	172413349	PIGC;PIGC;C1orf105	PIGC(-124)	Active TSS	− 0.069	0.030	**2.22E-02**	0.134	0.034	**7.78E-05**	0.200	0.045	**7.00E-06**	**3.88E-03**
cg10784067	chr16	14014435	ERCC4	ERCC4(+ 422)	Active TSS	− 0.127	0.032	**5.33E-05**	0.068	0.033	**3.78E-02**	0.201	0.045	**9.03E-06**	**5.01E-03**
cg14284055	chr1	24439399	MYOM3	MYOM3(-735)	Bivalent Enhancer	0.115	0.029	**8.28E-05**	− 0.066	0.037	7.12E-02	− 0.193	0.045	**1.94E-05**	**1.07E-02**
*cg18942110	chr15	91072797	CRTC3;CRTC3	CRTC3(-502)	Active TSS	0.031	0.030	3.06E-01	− 0.164	0.035	**2.23E-06**	− 0.187	0.045	**3.13E-05**	**1.74E-02**
cg09342330	chr14	89021109	PTPN21;PTPN21	PTPN21(-33)	Active TSS	0.045	0.030	1.31E-01	− 0.144	0.037	**8.30E-05**	− 0.189	0.046	**3.47E-05**	**1.93E-02**
cg21722170	chr6	31977443	TNXB;TNXA	C4B(-5095); C4A(+ 27643)	Quiescent/Low	0.034	0.030	2.53E-01	− 0.148	0.034	**1.57E-05**	− 0.183	0.045	**4.17E-05**	**2.31E-02**
cg03662217	chr21	35747882	FAM165B	SMIM11(-20)	Active TSS	0.039	0.029	1.85E-01	− 0.141	0.036	**7.27E-05**	− 0.184	0.045	**4.59E-05**	**2.55E-02**
cg15026265	chr12	28283812		PTHLH(-158910); CCDC91(-59566)	Quiescent/Low	− 0.038	0.029	1.95E-01	0.134	0.033	**6.58E-05**	0.176	0.044	**6.72E-05**	**3.73E-02**
cg00659421	chr7	86781760	DMTF1;DMTF1;DMTF1;DMTF1;DMTF1;DMTF1;DMTF1	DMTF1(-109)	Active TSS	− 0.050	0.031	1.08E-01	0.130	0.033	**7.53E-05**	0.178	0.045	**6.95E-05**	**3.86E-02**
*cg24917065	chr8	23418389	SLC25A37	SLC25A37(+ 32072); NKX3-1(+ 122050)	Quiescent/Low	− 0.135	0.030	**6.26E-06**	0.042	0.033	2.05E-01	0.174	0.044	**6.98E-05**	**3.87E-02**
*cg18281939	chr5	77783895	LHFPL2	SCAMP1(+ 127557); LHFPL2(+ 160752)	Weak transcription	− 0.022	0.029	4.55E-01	− 0.179	0.036	**6.26E-07**	− 0.177	0.045	**7.10E-05**	**3.94E-02**

### Enrichment analysis of sex-specific DNA methylation differences across genomic features

**Fig. 2:**
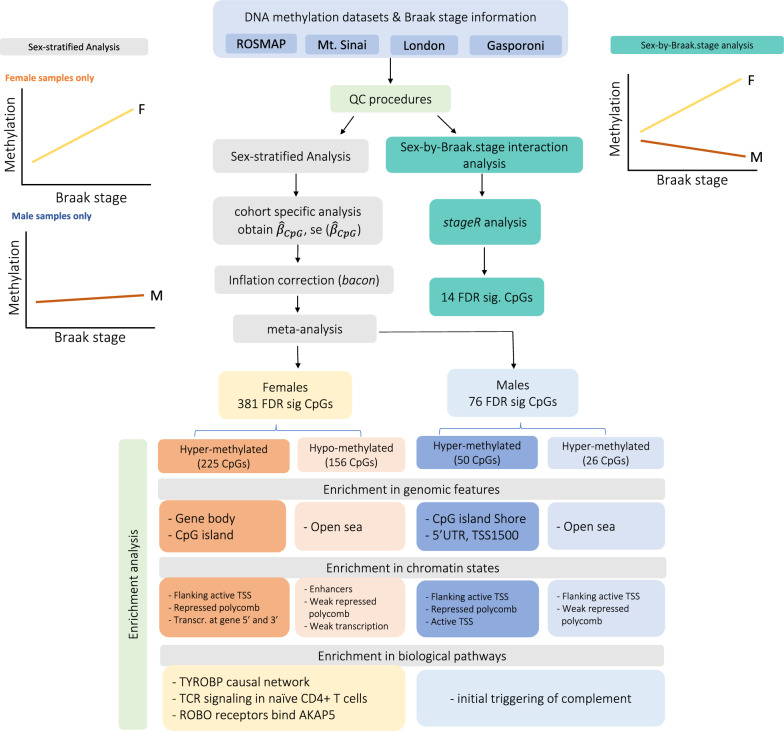
An overview of the sex-stratified analysis and sex-by-Braak stage interaction analysis. For each CpG, we performed two complementary analytical strategies, a sex-stratified analysis that examined methylation-Braak stage associations in male and female samples separately, and a sex-by-Braak stage interaction analysis that compared the magnitude of these associations between different sexes. At 5% FDR, the sex-stratified analysis identified 381 CpGs in the analysis of female samples and 76 CpGs in analysis of male samples, which were enriched in different biological pathways and genomic features

Figure 2 presents an overview of the enrichment analysis results. Compared to background probes, significant hypermethylated DMRs and CpGs in females are over-represented in CpG islands and gene bodies (Additional file 1: Fig. S3a, Additional file 2: Table S7). Significant hypermethylated DMRs and CpGs in males are over-represented in CpG island shores, 5′UTRs, and TSS1500s. In contrast, significant hypomethylated differences in females and males are over-represented in open seas (Additional file 1: Fig. S3b, Additional file 2: Table S7). These observations are consistent with the knowledge that during aging, human brain DNA methylation levels gradually increase (hypermethylation) at genomic loci located at CpG islands and gene promoters, whereas intergenic CpG sites are marked with hypomethylation [50, 51].

Our enrichment analysis for chromatin states showed that significant hyper-methylated differences in females were enriched in bivalent enhancer, flanking active TSS, repressed polycomb, and transcription at gene 5′ and 3′ regions (Additional file 1: Fig. S3c, Additional file 2: Table S8). On the other hand, significant hypermethylated differences in males were enriched in active TSS, flanking active TSS, and repressed polycomb regions. Hypomethylated differences in females were enriched in enhancers, weak repressed polycomb, and weak transcription regions, while hypomethylated differences in males were enriched in flanking active TSS and weak repressed polycomb regions (Additional file 1: Fig. S3d, Additional file 2: Table S8). Notably, the enrichment of hypermethylated differences in repressed polycomb regions in both female and male samples is consistent with our previous sex-combined meta-analysis, which also highlighted the enrichment of hyper-methylated Braak-associated DNA methylation differences in polycomb repressed regions [22].

Similarly, enrichment tests for regulatory elements using the LOLA software also supported the potential functional relevance of these significant differences in DNA methylation. Significant DMRs and CpGs in females and males were both enriched in binding sites of EZH2 and SUZ12 (Additional file 2: Table S9), which are subunits of polycomb repressive complex 2 (PRC2), consistent with the observed enrichment of methylation differences in PRC2 repressed regions (Additional file 1: Fig. S3c) and previous observations that DNA methylation often interact with PRC2 binding [52, 53]. PRC2 is a type of polycomb group (PcG) protein and plays important roles in multiple biological processes including proliferation and differentiation as well as maintenance of cellular identity through regulation of gene expression. Of particular relevance to AD, PRC2 silences genes involved in neurodegeneration and its deficiency leads to the de-repression of developmental regulators such as the Hox gene clusters, which manifest in progressive and fatal neurodegeneration in mice [54].

### Gene ontology and pathway analysis

Because of the relatively smaller number of gene sets being tested, a 25% FDR significance threshold, instead of the conventional 5% FDR, was suggested for GSEA [55]. At 25% FDR, the significant DNA methylation differences in females were enriched in TYROBP causal network (FDR = 0.014), TCR signaling in naïve CD4 + T cells (FDR = 0.130) and ROBO receptors bind AKAP5 (FDR = 0.160) gene sets, and significant methylation differences in males were enriched in the initial triggering of complement gene set (FDR = 0.245). The TYROBP causal network was previously inferred from a large-scale network analysis of human late-onset AD brains [56]; it was FDR significant (P-value < 0.001, FDR = 0.014) in females (Additional file 1: Fig. S4a), compared to a nominal association in males (P-value < 0.001, FDR = 0.620). Interestingly, the core enrichment subset of genes identified by GSEA in the female and male networks regulated by TYROBP involved DNA methylation differences at different genes (Additional file 1: Fig. S4b), highlighting different regulatory mechanisms for this gene network in males and females.

The comparison with gene ontology (GO) terms showed at 25% FDR, significant methylation differences in females were enriched in 25 GO biological processes (Table 4, Additional file 2: Table S10), many of which are involved in inflammatory responses associated with AD pathology including CD8 positive alpha beta T cell activation and interferon alpha production, as well as other biological processes critical for AD pathogenesis such as response to platelet derived growth factor and positive regulation of axon extension. For males, we did not identify any significant GO terms at 25% FDR; the strongest enrichment with nominal P-value less than 0.001 involved immune responses to the accumulation of amyloid-β (Aβ) in the brain, such as regulation of T cell activation via T cell receptor contact with antigen bound to MHC molecule on antigen presenting cell, and other biological processes recently implicated in AD such as response to angiotensin [57, 58] and cell redox homeostasis [59, 60].

### Correlation of sex-specific DNA methylation differences in AD neuropathology with expression levels of nearby genes

At 5% FDR, among FDR significant CpGs in females, all 381 CpGs were linked to a nearby gene by GREAT software, in which 14 were significantly associated with target gene expression levels (Additional file 2: Table S11), and half of them (n = 7) had effects in the negative direction. Among FDR significant CpGs in males, out of the 46 CpGs that were linked to a nearby gene, 2 were significantly associated with target gene expression and both were in the negative direction. Notably, in females, several of the most significant CpG methylation-gene expression associations were observed for the *HLA-DPA1* gene, which encodes microglia receptors involved in antigen presentation and is regulated by PU.1 [61]. In males, the most significant CpG-gene expression was for *HLA-DRB1*, another PU.1 target gene [61]. For the 14 CpGs identified by our sex-by-Braak stage interaction analysis, only one CpG (cg24917065) was significantly associated with target gene (*SLC25A37*) expressions.

### Correlation and overlap of sex-specific DNA methylation differences in AD neuropathology with genetic susceptibility loci

To evaluate if the significant methylation differences are located in the vicinity of sex-specific genetic variants implicated in AD, we compared our sex-specific CpGs and DMRs with the recently identified sex-specific SNPs associated with AD biomarkers [62] or AD pathology [63]. We found only 5 CpGs, mapped to the *SERP2, KCNE1, TNKS1BP1, FAM165B, PLCB4* genes were located within 500 kb of the sex-specific SNPs (Additional file 2: Table S12).

To search for mQTLs, which are genetic variants associated with DNA methylations, we next tested associations between the sex-specific CpGs and DMRs with SNPs that are located within 500 kb from them using 434 female samples, 254 male samples from the ROSMAP study, which had both genotype and DNA methylation data. While no mQTL-DMR pairs reached 5% FDR significance, we did identify 572 and 284 FDR-significant mQTL-CpG pairs associated with the sex-specific CpGs in females and males, respectively (Additional file 2: Tables S13, S14). Among the 381 and 76 sex-specific CpGs identified in female and male samples, respectively, 41 (11%) and 15 (20%) had at least one corresponding mQTL in brain samples. Among the 14 CpGs identified in our sex-by-Braak stage interaction analysis, 2 and 7 CpGs with at least one brain mQTL, corresponding to 21 and 236 significant mQTL-CpG pairs, were identified at 5% FDR in females and males, respectively (Additional file 2: Table S15). These mQTL-CpG pairs point to important potential molecular mechanisms of disease-associated genetic variants that might due, at least in part, to their influences on DNA methylations, which can be further validated in mechanistic studies.

### Drug target analysis of sex-specific DNA methylation differences

To investigate the clinical impact of the sex-specific DNA methylation differences, we next compared them with targets of drugs in the ChEMBL database [48] that are annotated to Alzheimer's disease, many of which are antipsychotic medications commonly prescribed to AD patients for treating psychiatric symptoms that accompany AD. We found that 13 CpGs and 2 DMRs, mapped to 20 genes, had overlap with targets of 16 different drugs (Additional file 2: Table S16). Among them, *CACNA1C* encodes a voltage-dependent calcium channel, which is a target of cholinesterase inhibitor donepezil. Previously, drug responses for donepezil were shown to be modulated by the sex hormone estrogen receptor alpha (ESR1) genotype [64]. Several CpGs and one DMR are mapped to targets of valproic acid, a mood stabilizer often prescribed for AD patients and was shown to have different pharmacokinetic profiles between male and female subjects [65]. Interestingly, two CpGs and 1 DMR also mapped to targets of caffeine, which was included in cocktail therapy in AD clinical trials [66, 67]. Although caffeine reduces the risk for AD [68, 69] in both men and women, the protective effect seems to be greater in women [70]. Also, *CHRM3* encodes muscarinic acetylcholine receptor, which is targeted by two commonly prescribed antipsychotic drugs for AD patients, trazodone and haloperidol. In both human and animal models, it has been observed treatment with haloperidol induces sex-specific DNA methylation differences [71, 72]. While this study could not establish the mechanisms at which DNA methylation interacts with drugs that AD patients take, we hypothesize that these might include the influence of DNA methylation on drug responses or the differences in DNA methylation resulted from drug actions.

## Discussion

To identify sex-specific differences in AD neuropathology, we performed a sex-stratified analysis and a sex-by-Braak stage interaction analysis for each cohort and then used a meta-analysis strategy to combine the cohort-specific association signals. In the sex-stratified analysis, as discussed above, a substantial number of the significant loci showed the same direction but attenuation of effect size for methylation-Braak stage association in a different sex (Tables 1, 2). Therefore, it is not surprising that many of these significant CpGs were identified previously in sex-combined meta-analysis [22]. Among FDR significant methylation differences in females, 325 CpGs (85%) and 40 DMRs (56%), mapped to genes such as *HOXA3, AZU1,* and *MBP* were also previously identified in our sex-combined meta-analysis [22] (Additional file 2: Tables S3, S5). Similarly, in the analysis of male samples, among FDR significant differences, 58 (76%) CpGs and 15 DMRs (56%), mapped to genes such as *MAMSTR, RHBDF2,* and *AGAP2,* overlapped with significant hits from sex-combined meta-analysis [22] (Additional file 2: Tables S4 and S6). However, our sex-specific analysis provided the new insight that the effects of these known AD genes appear to be predominately driven by effects in only one sex (Tables 1, 2).

On the other hand, our sex-specific analysis also uncovered novel methylation differences at 84 CpGs and 42 DMRs that were not identified previously by sex-combined analyses [22], which may had reduced power due to heterogeneity between the sexes. For example, among the top 10 CpGs in the sex-stratified analysis (Table 1), a new locus at cg22632947, which mapped to the gene body of the *PRKCA* gene, was highly significant in female samples (estimate = − 0.139, P-value = 1.50 × 10^–7^, FDR = 3.00 × 10^–3^), but not significant in male samples (estimate = − 0.005, P-value = 0.857, FDR = 0.995) (Additional file 1: Fig. S5). The *PRKCA* gene encodes protein kinase Cα (PKCα), which participates in synaptic loss resulting from the accumulation of amyloid-β (Aβ) in AD neuropathology [73, 74]. Another novel locus is at cg18942110 in the promoter of the *CRTC3* gene, where methylation-Braak stage association was highly significant in male samples (estimate = − 0.164, P-value = 2.23 × 10^–6^, FDR = 3.19 × 10^–2^), but not significant in female samples (estimate = − 0.031, P-value = 0.306, FDR = 0.952) (Additional file 1: Fig. S5). CRTC3 is a member of the CRTC family, which are coactivators of the transcription factor CREB (cAMP-response element binding protein). In addition to its crucial role in maintaining synaptic plasticity and facilitation of short-term memory to long-term memory, the CREB signaling pathway also mediates synapse loss induced by Aβ in AD [75]. Notably, synapse loss significantly correlates with cognitive impairment [76, 77] and has been observed to be an early feature of AD pathogenesis [78, 79].

The sex-by-Braak stage interaction analysis also uncovered several additional novel methylation loci that affected AD neuropathology in a sex-specific manner. Notably, none of the 14 CpGs detected in our interaction analysis was identified in previous large-scale DNA methylation studies [18–22], suggesting that sex-specific differences such as these can be missed by conventional studies that do not consider the impact of sex. This is likely due to the cancelation of effects in sex-combined analysis because the majority of these 14 CpGs had different directions of methylation-Braak stage effects in male and female samples (Table 3). Among genes mapped to these 14 CpGs, TMEM39A is a member of the transmembrane (TMEM) protein family. In recent GWAS, a genetic variant on *TMEM39A* was discovered and replicated as an important risk locus for multiple sclerosis, an autoimmune condition of the central nervous system [80, 81]. While relatively little is known about the role of *TMEM39A* in AD, given its important contributions to inflammation, dysregulated type I interferon responses, and other immune processes [82] which are also implicated in AD, methylation differences affecting this gene are particularly relevant. Another noteworthy gene is *TNXB* and its pseudogene *TNXA*, which are located in the major histocompatibility complex (MHC) class III region on chromosome 6. *TNXB* encodes tenascin proteins, which are extracellular matrix glycoproteins demonstrated to modulate synaptic plasticity in the brain [83]. In particular, genetic variants at the *HLA-DQB1* locus discovered in the recent AD genetic meta-analysis [84] included eQTLs for *TNXB/TNXA* in brain tissues [84, 85].

Consistent with previous studies [18, 19, 86, 87], we observed the majority of these sex-specific differences were hyper-methylated in samples with AD neuropathology, for which methylation levels increased as the AD Braak stage increased (Additional file 2: Tables S17, S18). More specifically, 59% of the significant CpGs and 69% of the significant DMRs in females, along with 66% of the significant CpGs and 89% of the significant DMRs in males were hyper-methylated in samples with AD neuropathology (Additional file 2: Tables S3–S6).

To better understand the relevance of these Braak-associated sex-specific differences, we also compared our results with several previous studies. The comparison with Xia et al. [16] and Xu et al. [17], which examined differential methylation between males and females in the prefrontal cortex, but without considering AD neuropathology [16, 17], showed our results were largely distinct. Among 451 unique CpGs identified in our sex-stratified analysis or sex-by-Braak stage interaction analysis, only 16 were also identified in Xia et al. [16] and none were identified in Xu et al. [17] (Additional file 2: Tables S3–S6). This is probably due to different hypotheses tested in our study and the sexual dimorphism studies – while our study examined the impact of sex on methylation-Braak stage association, the previous studies examined differential methylation between the sexes, regardless of AD neuropathology. The comparison of our results with sex-specific DNA methylation differences in fetal brain development [88, 89] also showed very little overlap (Additional file 2: Table S19); one hypothesis could be that the Braak-associated sex-specific DNA methylation differences identified in this study might be influenced by environmental risk factors for AD, such as diet and exercise.

The results of our gene set analysis highlighted a number of critical sex-specific biological processes in AD neuropathology. Notably, the TYROBP causal network reached the FDR significance threshold in females (FDR = 0.014) but was only nominally significant in males. Interestingly, Braak-associated CpG methylation differences that drove pathway associations (core enrichment genes) occurred at different genes in females and males (Additional file 1: Fig. S4), indicating a potentially sex-specific regulatory mechanism for this network. TYROBP (TYRO protein tyrosine kinase-binding protein) is a key regulator of the complement pathway in the immune/microglia network, which is activated as Aβ accumulates in LOAD brains [56, 90]. TYROBP is a transmembrane adaptor protein for TREM2, SIRPβ1, and CR3 receptors, which are known to be involved in AD pathogenesis [90–92]. Also, TYROBP is regulated by *SPI1*, a central hub for the network of genes involved in myeloid immune response in neurodegeneration [93]. In patients with LOAD, TYROBP was observed to be up-regulated in the brains in multiple cohorts [56]. Recent studies suggested TYROBP-mediated signaling is involved in multiple important functions as aggregating Aβ activates microglia, including enhanced phagocytosis of damaged neurons [56, 90] and suppression of inflammatory responses [94], as well as neuronal pruning activity [56]. Interestingly, in gene ontology (GO) analysis, among the most significant GO Biological Process terms (P-value < 0.001) in females and males, none of them overlapped (Additional file 2: Table S10), even though the relevancy of all the top biological processes were supported by recent AD literature (Table 4). These results suggest different biological processes are associated with AD pathology in males and females.

**Table 4:** Top 10 most significant GO Biological processes enriched with sex-specific DNA methylation differences associated with AD Braak stage in females and males. Shown are GSEA results including the number of genes in the gene set (SIZE), normalized enrichment score (NES), P-value, FDR, and relevant AD literature for the gene set

Gene Set	SIZE	NES	P-value	FDR	Relevance to AD
*Top 10 GO Biological Process terms in females*					
INTEGRIN_ACTIVATION	24	2.105	0	9.53E−02	Wennstrome et al. [98]
RESPONSE_TO_PLATELET_DERIVED_GROWTH_FACTOR	19	2.116	0	1.07E−01	Sil et al. [99]
I_KAPPAB_PHOSPHORYLATION	19	2.081	0	1.13E−01	Jha et al. [100]
NEGATIVE_REGULATION_OF_INTERLEUKIN_8_PRODUCTION	18	2.133	0	1.21E−01	Qin et al. [101]
POSITIVE_REGULATION_OF_MACROPHAGE_MIGRATION	25	2.048	0	1.46E−01	Bacher et al. [102]
TOLL_LIKE_RECEPTOR_SIGNALING_PATHWAY	142	2.031	0	1.61E−01	Fiebich et al. [103]
NEGATIVE_REGULATION_OF_TUMOR_NECROSIS_FACTOR_SUPERFAMILY_CYTOKINE_PRODUCTION	57	2.007	0	1.90E−01	Chang et al. [104]
REGULATION_OF_SYNCYTIUM_FORMATION_BY_PLASMA_MEMBRANE_FUSION	28	1.944	0	1.98E−01	Armoto et al. [105]
RESPONSE_TO_VITAMIN_A	19	1.945	0	2.08E−01	Ono et al. [106]
POSITIVE_REGULATION_OF_AXON_EXTENSION	42	1.931	0	2.08E−01	Kanaan et al. [107]
*Top 10 Biological Process terms in males*					
REGULATION_OF_T_CELL_ACTIVATION_VIA_T_CELL_RECEPTOR_CONTACT_WITH_ANTIGEN_BOUND_TO_MHC_MOLECULE_ON_ANTIGEN_PRESENTING_CELL	6	1.869	0	5.98E−01	Schetters et al. [108]
REGULATION_OF_SYSTEMIC_ARTERIAL_BLOOD_PRESSURE_BY_CIRCULATORY_RENIN_ANGIOTENSIN	18	1.964	0	6.11E−01	Cosarderelioglu et al. [57]
NEGATIVE_REGULATION_OF_REACTIVE_OXYGEN_SPECIES_BIOSYNTHETIC_PROCESS	29	1.745	0	6.12E−01	Manoharan et al. [59]
COMPLEMENT_ACTIVATION	68	1.741	0	6.26E−01	Morgan [97]
RESPONSE_TO_ANGIOTENSIN	26	1.746	0	6.36E−01	Benigni et al. [58]
CELL_REDOX_HOMEOSTASIS	55	1.721	0	6.49E−01	Chen et al. [60]
PROTEIN_DEMETHYLATION	28	1.832	0	6.77E−01	Esposito et al. [109]
IMMUNE_RESPONSE_INHIBITING_CELL_SURFACE_RECEPTOR_SIGNALING_PATHWAY	6	1.781	0	7.02E−01	Schetters et al. [108]
DICARBOXYLIC_ACID_CATABOLIC_PROCESS	17	2.052	0	7.57E−01	Griffin et al. [110]
GLUTAMINE_FAMILY_AMINO_ACID_METABOLIC_PROCESS	70	1.657	0	7.75E−01	Conway et al. [111]

Importantly, a number of these sex-specific biological processes pointed to important potential biomarkers and therapeutic targets for the treatment of AD. For example, one of the top biological processes enriched with significant methylation differences in female samples is response to platelet derived growth factor. Recently, multiple studies have shown that reduced levels of platelet-derived growth factors (PDGFs) in plasma significantly correlate with mild cognitive impairment and have proposed PDGFs as a potential biomarker for AD [95, 96]. For the significant methylation differences in male samples, one of the top biological processes highlighted by our enrichment analysis is dysregulation in the complement system. Recently, a number of novel agents targeting the complement system are being developed and tested in clinical trials for potential effective therapy for AD [97]. Therefore, clinical trials testing potential treatment for AD patients might have more power for detecting treatment effects by considering sex and targeting the subgroup with the higher predicted benefit based on patient molecular profiles such as DNA methylation.

There are several limitations for this study. The methylation levels in the studies analyzed here were measured on the bulk prefrontal cortex, which contains a complex mixture of cell types. To reduce confounding effects due to cellular heterogeneities, we included the estimated neuron proportion of each brain sample as a covariate variable in all our analyses. Currently, a challenge with cell-type-specific studies is that they are often limited to smaller sample sizes due to labor-intensive sample preparation procedures and therefore have limited statistical power. Also, we did not identify any CpGs or DMRs from chromosome X, this might suggest that sex differences in AD neuropathology are not primarily due to chromosome X. Alternatively, the lack of association might also be due to the limited coverage by the 450k array. Future studies utilizing high throughput sequencing that provides better coverage of the epigenome will help clarify the role of the X chromosome in AD neuropathology. Finally, the associations we identified in this study do not necessarily reflect causal relationships. Future studies that employ longitudinal designs are needed to identify causal changes in DNA methylation as AD initiates and progresses.

In summary, our study highlighted the importance of stratifying on sex and analyzing sex-by-disease interaction in the analysis of DNA methylation data to discover the epigenetic architectures underlying AD neuropathology. Our meta-analysis discovered many novel sex-specific DNA methylation differences consistently associated with the AD Braak stage in multiple studies. Because of cancelation of effects in different directions, or dilution from samples with no effect, these sex-specific effects would be missed by sex-combined analysis. Moreover, for many genes previously linked to AD neuropathology, our work provided evidence that the DNA methylation differences at these genes were predominately driven by effects in only one sex. Our enrichment analysis highlighted divergent biological processes in males and females, which underscored sex-specific regulatory mechanisms involved in AD neuropathology. Finally, our results also have important implications for precision medicine—many of the sex-specific DNA methylation differences also pointed to important potential AD biomarkers and therapeutic targets, suggesting a pressing need for developing and applying sex-specific treatment strategies for AD.

## Supplementary Information

**Additional file 1:** Supplementary Figures.** Figure S1.** Quantile-quantile (QQ) plots of observed and expected distributions of P-values in Gasparoni, London, Mount Sinai, and ROSMAP cohorts.** Figure S2.** Comparison of methylation-Braak stage associations in female samples and male samples.** Figure S3.** Enrichment of FDR-significant CpGs and CpGs located within FDR-significant DMRs with positive and negative effect estimates in various genomic features and chromatin states.** Figure S4.** Gene Set Enrichment of the TYROBP causal network with sex-specific Braak-associated DNA methylation differences.** Figure S5.** Forest plots for several top CpGs identified in sex-stratified analysis and sex-by-Braak stage interaction analysis.

**Additional file 2:** Supplementary Tables.** Table S1.** Sample information of the brain samples datasets included in the meta-analysis.** Table S2.** Quality control (QC) information on DNA methylation samples and probes for each cohort contributing to this meta-analysis.** Table S3.** In females, a total of 381 differentially methylated CpGs were significantly associated with the AD Braak stage at 5% FDR in the meta-analysis of four brain samples cohorts (Gasparoni, London, Mt. Sinai, ROSMAP).** Table S4.** In males, a total of 76 differentially methylated CpGs were significantly associated with the AD Braak stage at 5% FDR in the meta-analysis of four brain samples cohorts (Gasparoni, London, Mt. Sinai, ROSMAP).** Table S5.** In females, at 5% FDR, a total of 72 co-methylated DMRs were significantly associated with AD Braak stage in the meta-analysis of the four brain samples cohorts.** Table S6.** In males, at 5% FDR, a total of 27 co-methylated DMRs were significantly associated with AD Braak stage in the meta-analysis of four brain samples cohorts.** Table S7.** Enrichment of sex-specific DNA methylation changes in different genomic features.** Table S8.** Enrichment of sex-specific DNA methylation changes in different chromatin states.** Table S9.** Enrichment of sex-specific DNA methylation changes in binding sites of transcription factors and chromatin proteins assayed by ENCODE and CODEX projects.** Table S10.** Results of Gene Set Enrichement analysis of sex-specific CpGs and DMRs in female and males.** Table S11.** Significant association between sex-specific differences and expression levels of nearby genes.** Table S12.** The sex-specific CpGs and DMRs located within 500 kb of sex-specific SNPs associated with AD biomarkers or AD neuropathology.** Table S13.** mQTLs assoicated with FDR-significant CpGs in females.** Table S14.** mQTLs assoicated with FDR-significant CpGs in males.** Table S15.** mQTLs associated with significant CpGs identified by sex-by-Braak stage interaction analysis.** Table S16.** Sex-specific DNA methylation differences overlapped with AD drug targets in ChEMBL database.** Table S17.** Average beta values at each Braak stage for significant CpGs identified in female samples.** Table S18.** Average beta values at each Braak stage for significant CpGs identified in male samples.** Table S19.** Overlap of Braak-associated sex-specific CpGs and DMRs with sex-specifc DNA methylation changes in fetal development.

## Data Availability

All datasets analyzed in this study are publicly available as described in Additional file 2: Table S1. The Mt. Sinai, London, Gasparoni, and ROSMAP datasets can be accessed from GEO (accessions GSE80970, GSE59685, GSE66351) and Synapse (https://doi.org/10.7303/syn3157275). The scripts for the analysis performed in this study can be accessed at https://github.com/TransBioInfoLab/ad-meta-analysis-by-sex.
