# Supplementary material for: Sex-specific DNA methylation differences in Alzheimer’s disease pathology
Source: Acta Neuropathol Commun. 2021 Apr 26;9:77. doi: 10.1186/s40478-021-01177-8 (PMC8074512; doi:10.1186/s40478-021-01177-8)

Figure S1 Quantile-quantile (QQ) plots of observed and expected distributions of p-values in Gasparoni, London, Mount Sinai, and ROSMAP cohorts. λ is the genomic inflation factor, and λ_{bacon} is the genomic inflation factor estimated using the method of Iterson et al. (2017) (PMID: 28129774), as implemented in the bacon R package. Shading indicates 95% confidence intervals. Reference line in red indicates expected distribution of $-\log_{10}$(P-values) under the null hypothesis of no association.

(A) for analysis results of female samples

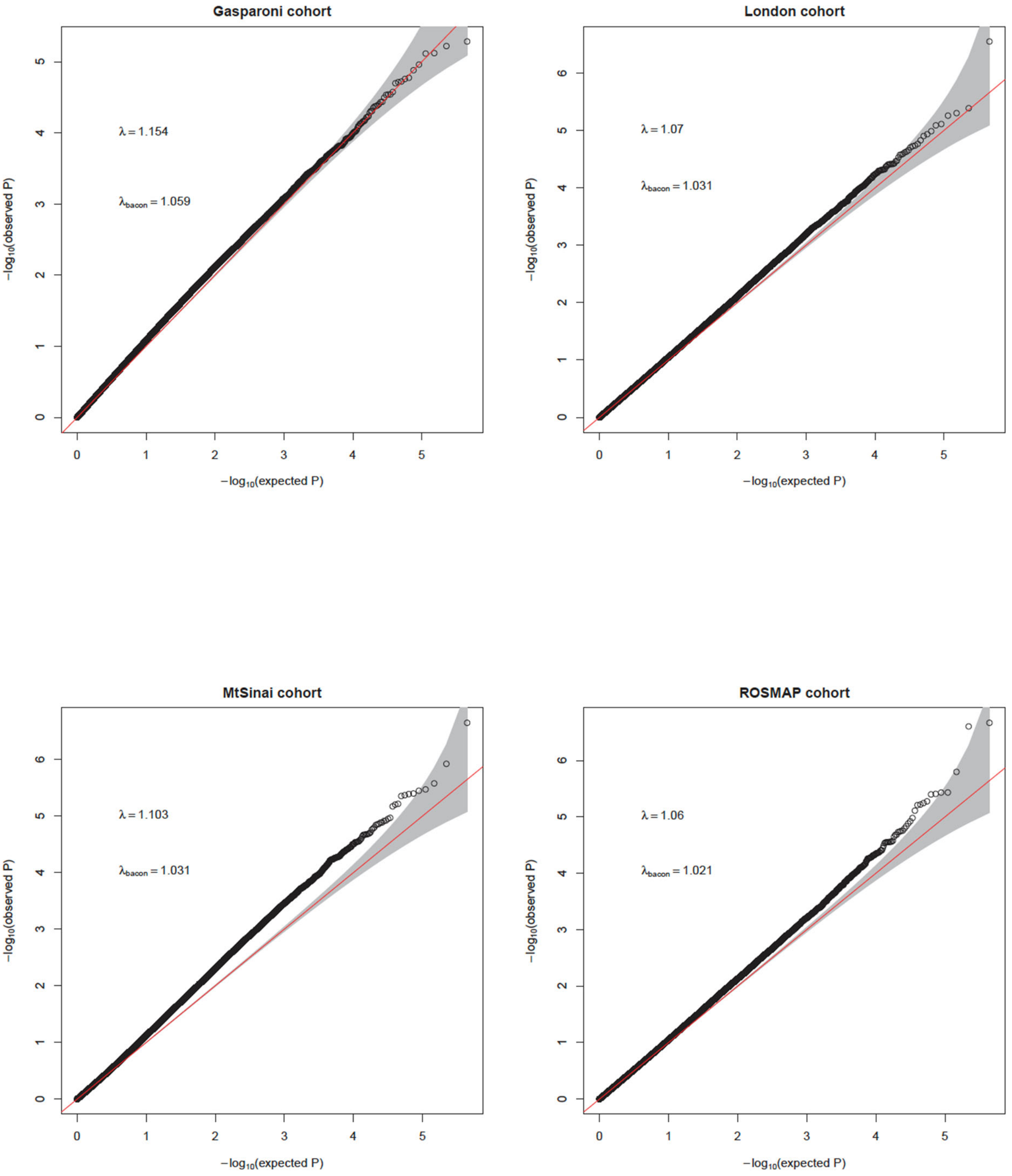

(B) for analysis results of male samples

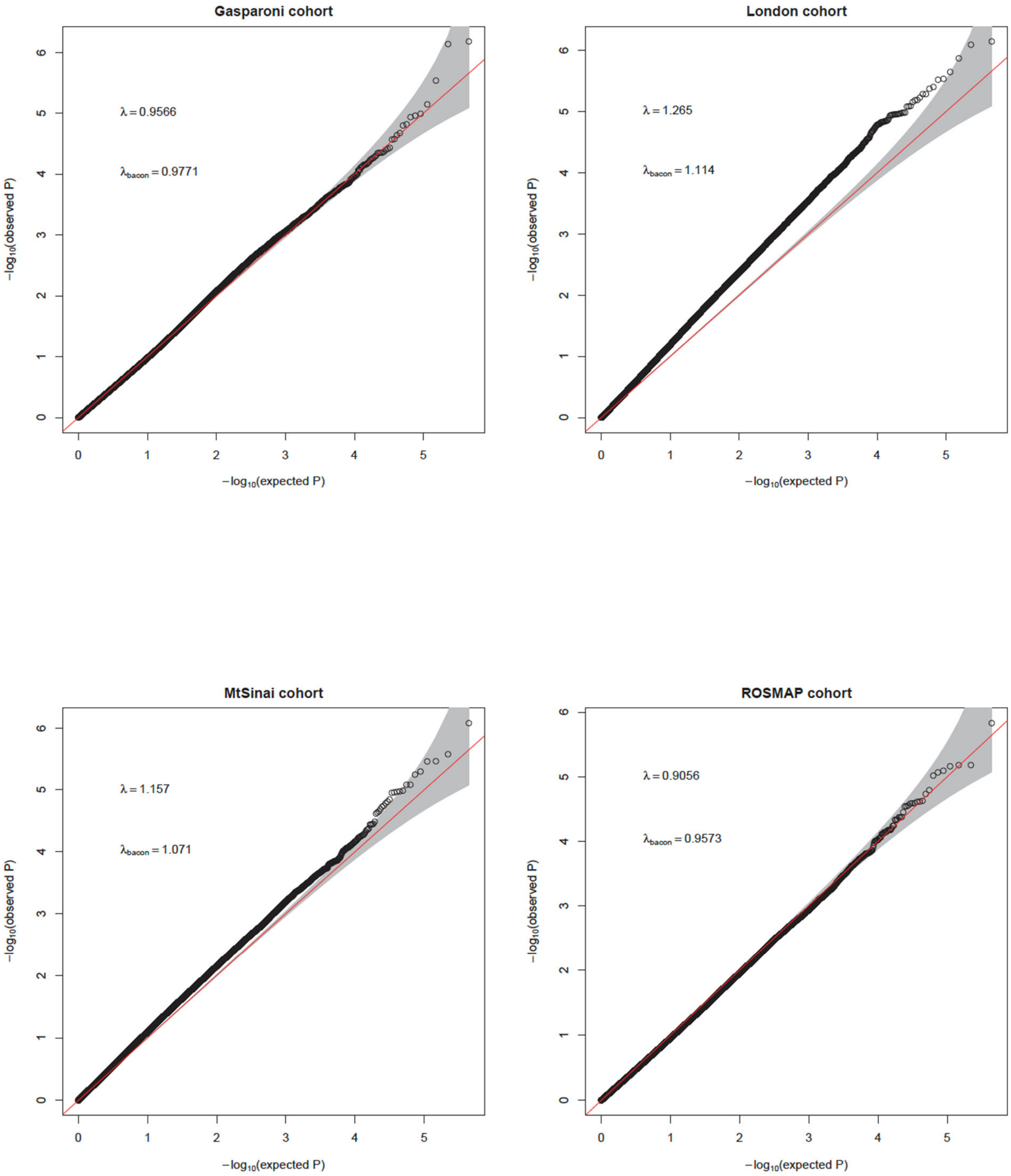

Figure S2 Comparison of methylation-Braak stage associations in female samples and male samples.

(A) Comparison of effect estimates for methylation-Braak stage associations obtained in female samples analysis vs. those in male samples analysis.

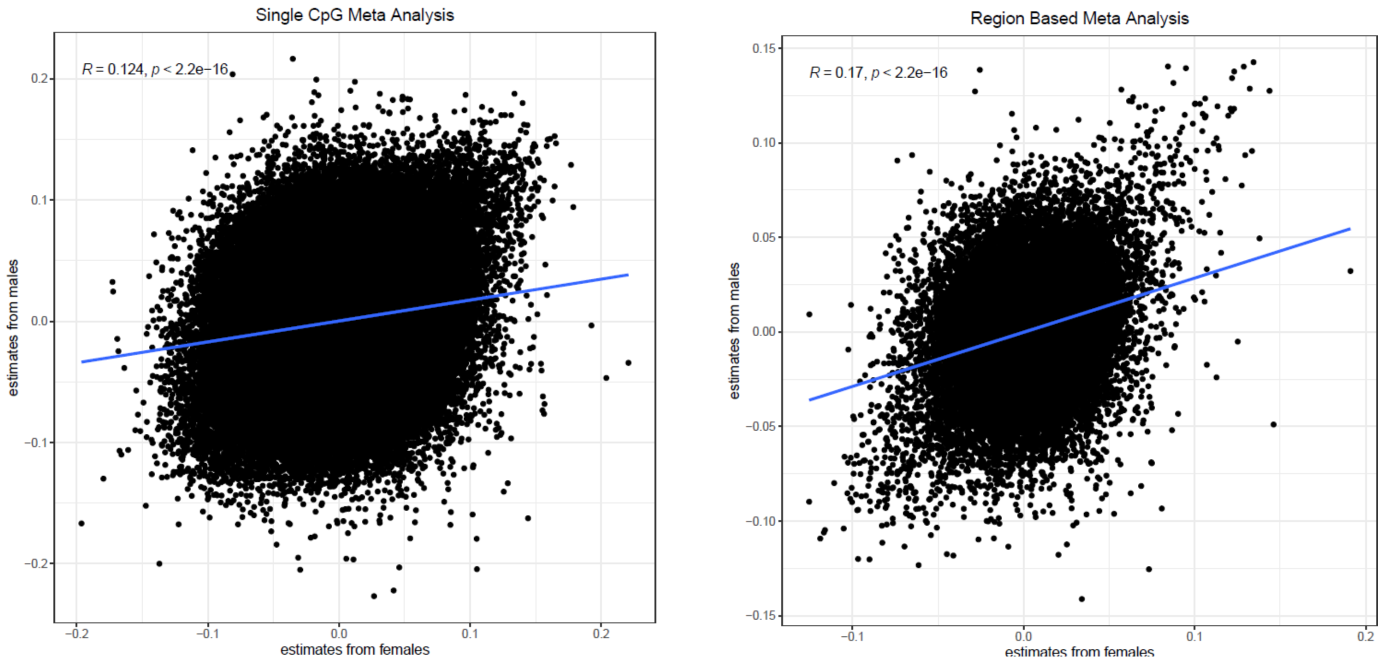

(B) Comparison of -$\log_{10}$ P-values for methylation-Braak stage associations obtained in female samples analysis vs. those in male samples analysis.

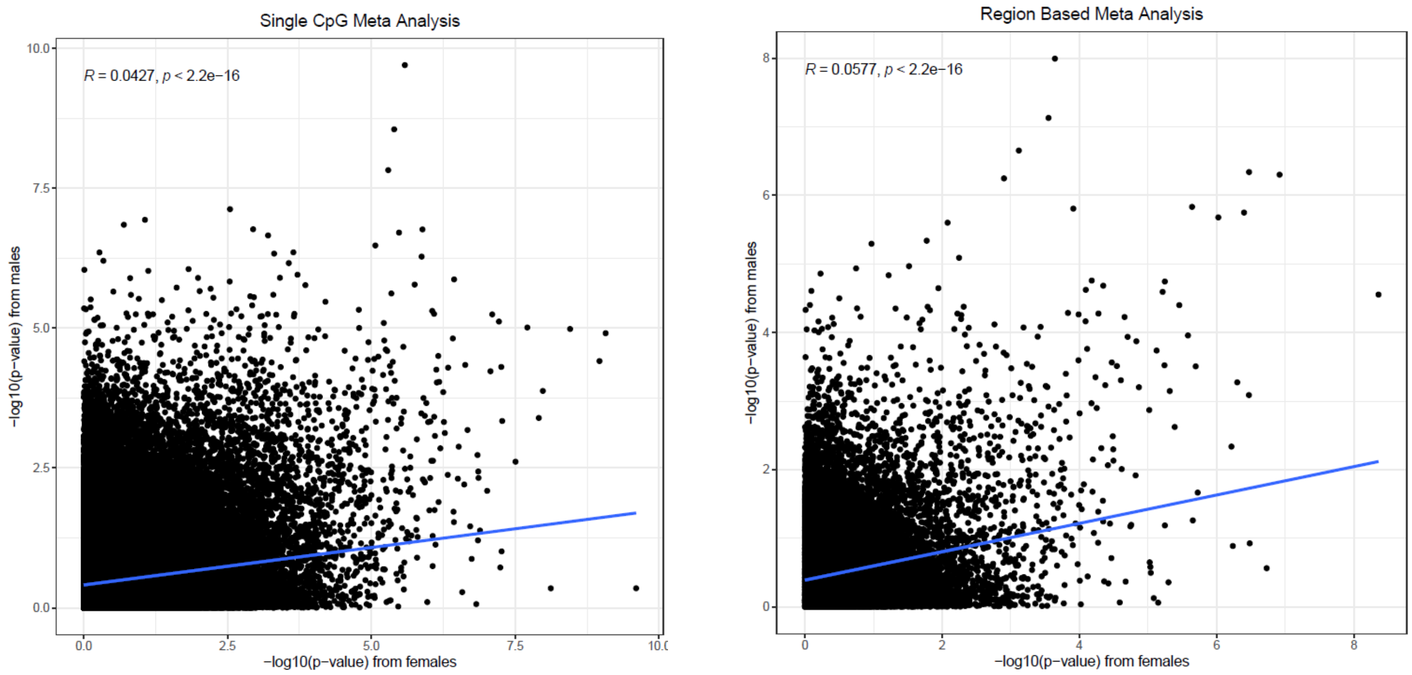

Figure S3 Enrichment of FDR significant CpGs and CpGs located within FDR significant DMRs with positive and negative effect estimates in various (A) (B) genomic features and (C) (D) chromatin states. *** indicates P-value < 0.001, ** indicates P-value < 0.01, * indicates P-value < 0.05

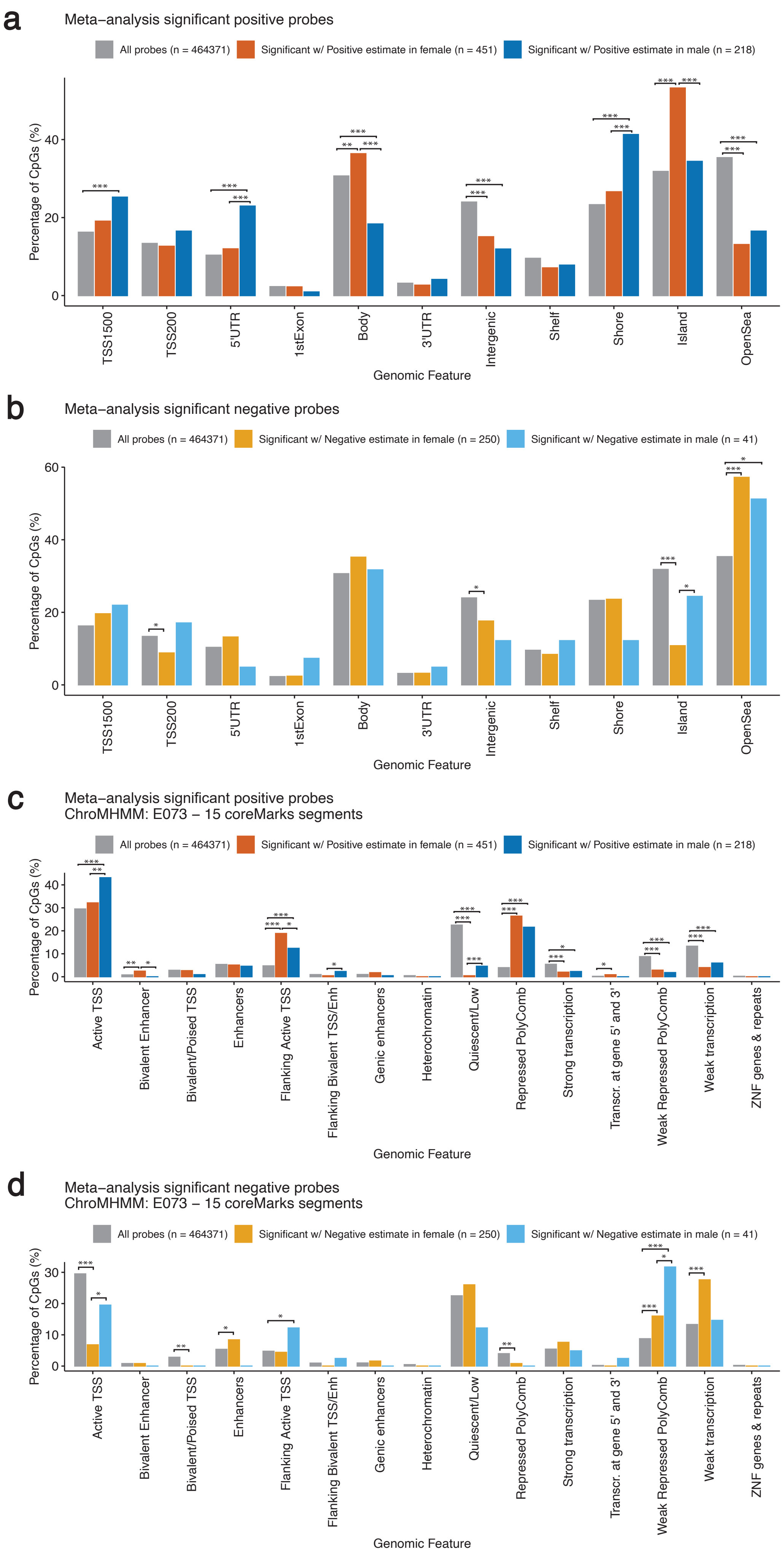

Figure S4 The TYROBP causal network.

(a) Genes with significant DNA methylation changes in female samples are enriched in the TYROBP causal network (FDR = 0.014).

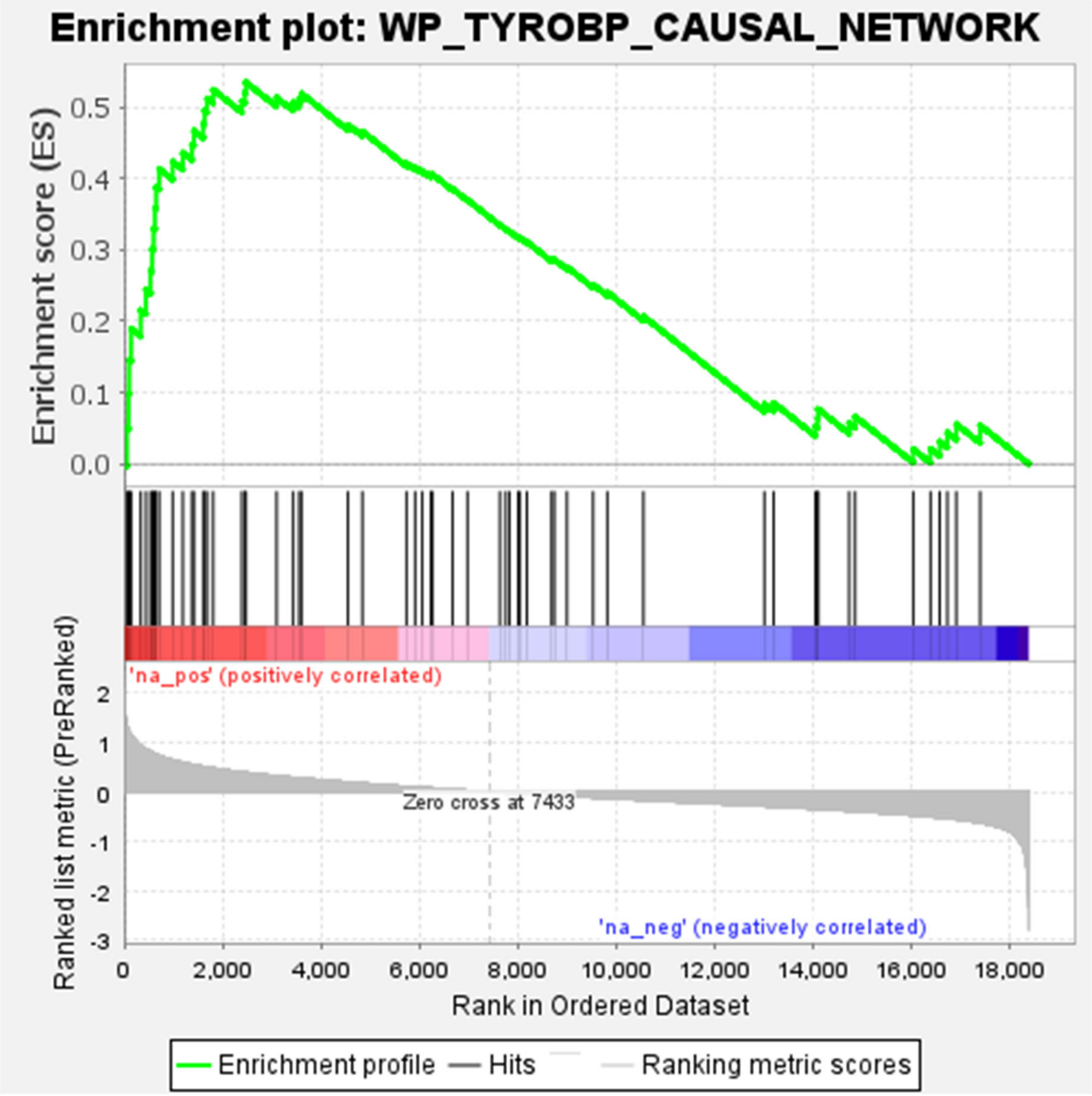

(b) Orange = core enrichment genes identified by GSEA in the analysis of female samples, blue = core enrichment genes identified by GSEA in the analysis of male samples, and green = core enrichment genes identified by GSEA in both male and female samples analysis.

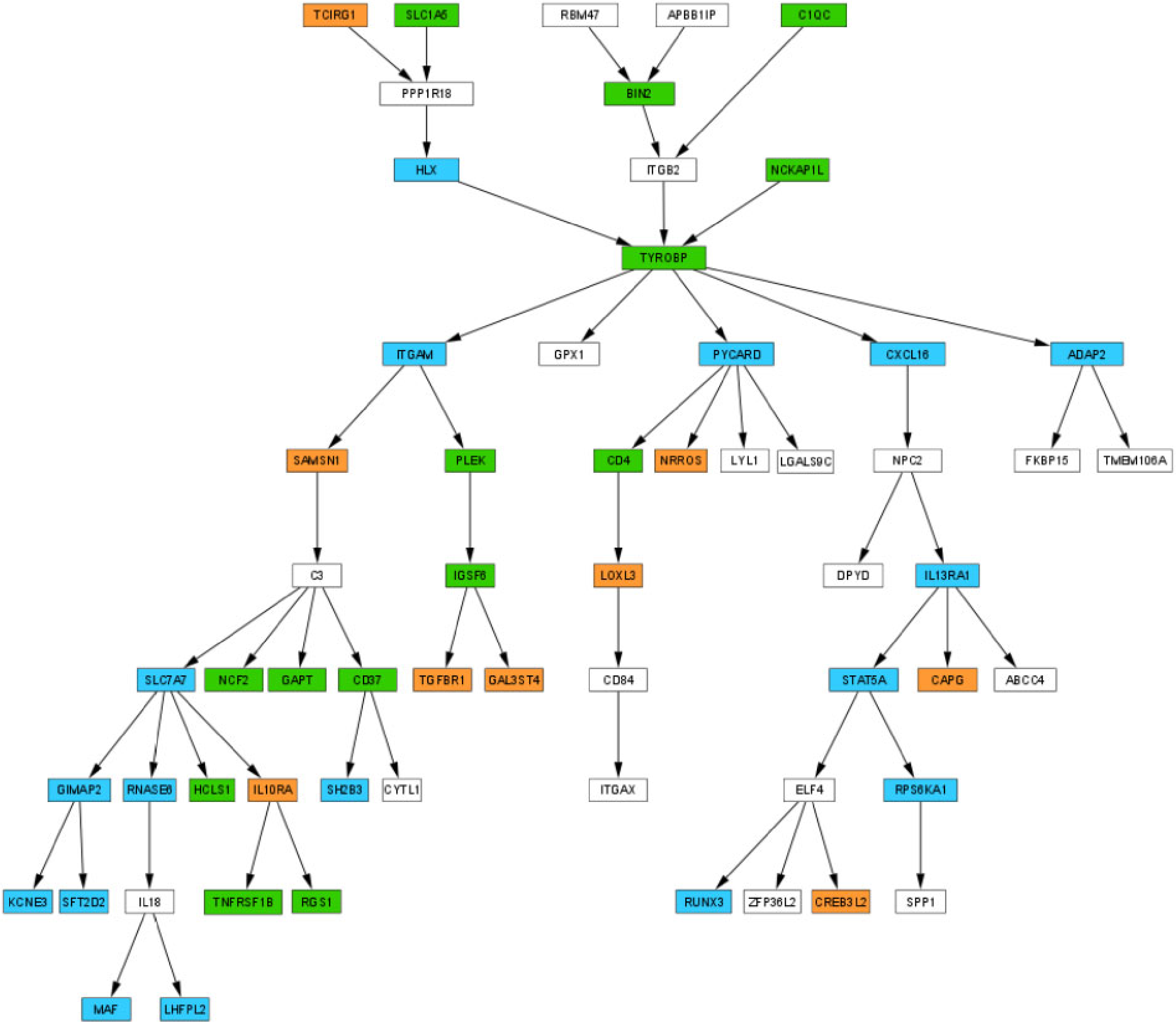

Figure S5 Forest plots for example top CpGs identified in sex-stratified analysis and sex-by-Braak stage interaction analysis.

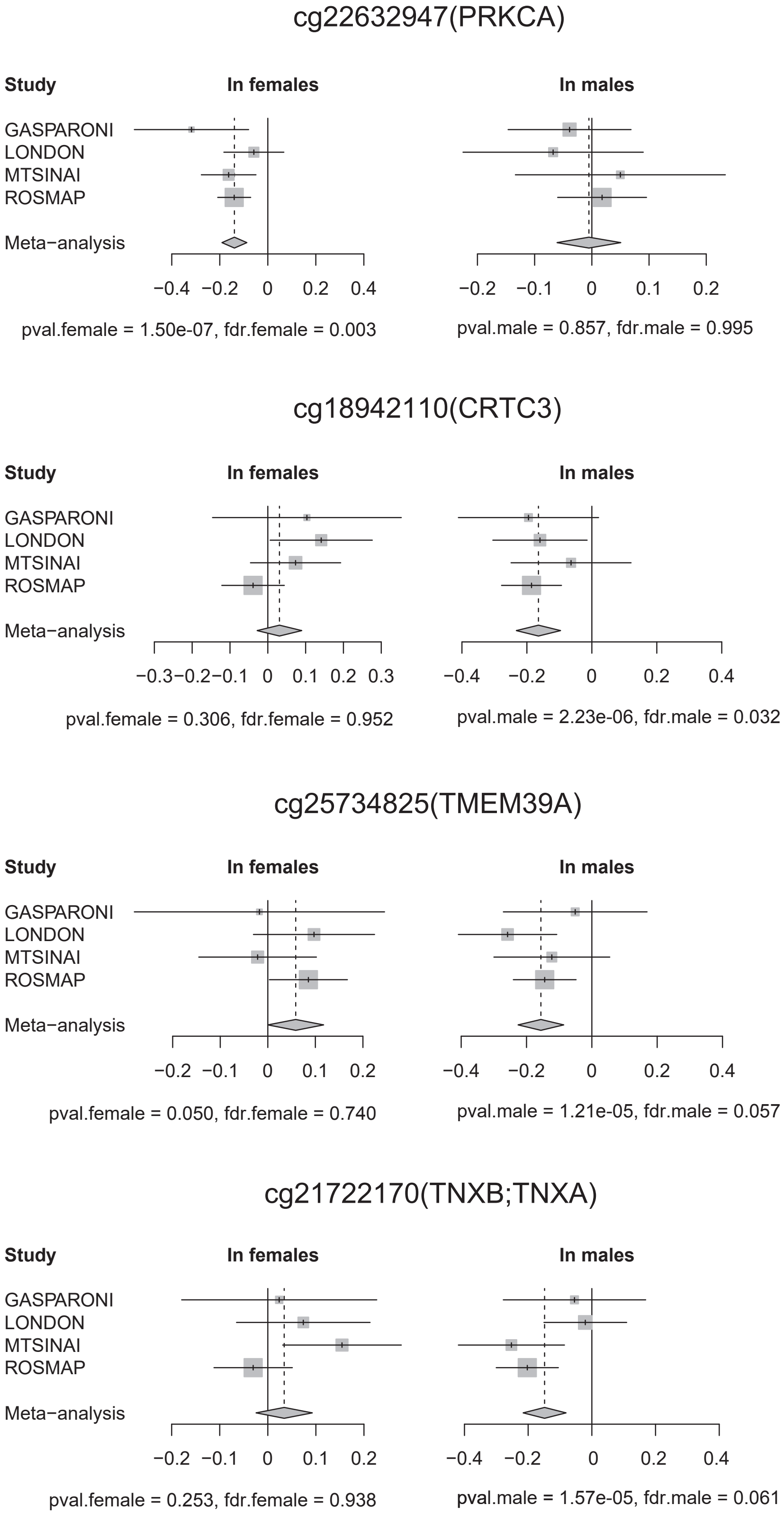

Supplement: Supplementary file 1 — Additional file 1: Supplementary Figures. Figure S1. Quantile-quantile (QQ) plots of observed and expected distributions of P-values in Gasparoni, London, Mount Sinai, and ROSMAP cohorts. Figure S2. Comparison of methylation-Braak stage associations in female samples and male samples. Figure S3. Enrichment of FDR-significant CpGs and CpGs located within FDR-significant DMRs with positive and negative effect estimates in various genomic features and chromatin states. Figure S4. Gene Set Enrichment of the TYROBP causal network with sex-specific Braak-associated DNA methylation differences. Figure S5. Forest plots for several top CpGs identified in sex-stratified analysis and sex-by-Braak stage interaction analysis. [file 40478_2021_1177_MOESM1_ESM.pdf]
